# Enhancing Pig Behavior Recognition in Complex Environments: A Transfer Learning-Assisted YOLO11 Network with Wavelet Convolution and Synergistic Attention

**DOI:** 10.3390/ani16060964

**Published:** 2026-03-19

**Authors:** Taoyang Wang, Yu Hu, Hua Yin

**Affiliations:** School of Software, Jiangxi Agricultural University, Nanchang 330045, China; 19307026819@163.com (T.W.); 18779898658@163.com (Y.H.)

**Keywords:** pig behavior recognition, YOLO11, attention mechanism, transfer learning, lightweight detection

## Abstract

Pig behavior recognition plays a vital role for early disease detection, animal welfare evaluation, and precision agriculture. Current deep learning methods tend to be complex, parameter intensive, or lack generalization in unstructured farming scenarios, hindering their deployment on resource-limited devices. To address this issue, we propose three optimizations based on the lightweight YOLO11n: (1) embed SCSA-CBAM in C3k2 layers to enhance multi-scale feature discrimination; (2) introduce WFU in the neck for dynamic cross-scale feature integration; and (3) replace standard convolutions in the backbone with WTConv to reduce the computational overhead. Initialized with COCO pre-trained weights, the proposed model employs a two-stage transfer learning approach combined with data augmentation. On a self-built six-category pig behavior dataset based on public datasets of 2480 original images (split into training/validation sets at an 8:2 ratio via stratified random sampling), the optimized YOLO11n-SCSA-WFU-WT achieves an mAP@0.5 of 0.974 and mAP@0.5:0.95 of 0.785, with 3.40 M parameters, 7.8 GFLOPs, and 72.28 FPS, while achieving substantial accuracy improvements over the baseline and maintaining lightweight performance over the baseline. Ablation experiments verify the independent contributions of each module, and comparisons with mainstream models demonstrate a more favorable accuracy–efficiency trade-off. The overall results confirm the effectiveness of our method, which facilitates real-time pig behavior detection in future smart livestock management.

## Simple Summary

Pigs’ behavioral changes are important indicators of their health and welfare. Traditional manual monitoring is inefficient, labor intensive, and lacks scalability for continuous real-time livestock management. This study targets six core pig behaviors covering feeding, resting, activity, and behaviors associated with physiological metabolism. We propose a lightweight and high-precision pig behavior recognition system based on YOLO11n, combined with Spatial Cross-Scale Attention (SCSA), a Weighted Feature Fusion Unit (WFU), and Wavelet Transform Convolution (WTConv). We adopt a two-stage transfer learning strategy: freezing the backbone during initial training, and then fine-tuning the full network. Experiments show that the model achieves 97.4% mAP@0.5 and 72.28 FPS, balancing lightweight characteristics and recognition accuracy. This system supports intelligent pig farming and animal welfare monitoring, offering a practical solution for smart livestock management.

## 1. Introduction

The swine sector is a core component of China’s livestock industry, serving a critical function in upholding food security and optimizing residents’ dietary patterns [1]. As large-scale intensive farming expands, there is an urgent need for long-lasting, non-interrupted, and low-cost pig behavior monitoring to underpin herd health supervision, precision feeding, and animal welfare evaluation [2].

Traditional pig behavior-monitoring approaches include manual observation and wearable device-based methods. Manual observation is subjective and inefficient, and it is challenging to achieve 24 h continuous monitoring [3]. Wearable devices such as RFID tags and accelerometers are reliable in specific scenarios, but usually incur relatively high hardware costs and complex deployment and maintenance on large-scale farms. Compared with these two approaches, non-contact computer vision-based methods show unique advantages in practical farming: they do not require additional wearable devices, cause no interference to pigs, and are more suitable for low-cost, large-scale, and long-term automatic monitoring. As pigs’ behavioral changes are direct indicators of their health and welfare—abnormal patterns often signal diseases, environmental stress, or management issues—establishing an intelligent automated monitoring system is critical [4]. This study focuses on six core behaviors (standing, walking, prone lying, lateral lying, eating, and drinking), which comprehensively cover the “feeding–resting–activity–physiological needs” dimensions in the daily lives of pigs [5].

Advancements in computer vision and deep learning have promoted non-contact behavior recognition [6]. Conventional machine learning methods (e.g., background subtraction and SVM) lack robustness in complex farm environments (e.g., lighting variations and occlusions) [7]. In this study, we characterize “complex environments” by three specific challenges: (1) severe inter-animal occlusion due to high-density stocking; (2) uncontrolled lighting conditions ranging from strongly backlight to low illumination; and (3) high background clutter where textures resemble pig bodies. Subsequent deep learning detectors (e.g., Faster R-CNN and YOLOv5) have improved accuracy but suffer from parameter redundancy and poor adaptability to resource-constrained edge devices [8]. Recent lightweight models have made progress: for example, a multi-scenario pig behavior recognition model based on YOLOv8n significantly improves the detection accuracy of standing, prone lying, lateral lying and feeding behaviors in complex lighting and environments by introducing SPD-Conv, LSK attention and a small target detection head, while taking into account the lightweight deployment requirements [9]. YOLO-DLHS-P combines large-kernel convolutions, LSKA attention mechanisms, HWD downsampling and Shape-IoU loss based on YOLOv8n, and achieves substantial model compression through pruning, reducing parameters and GFLOPs while ensuring accuracy, and is suitable for real-time behavior recognition on embedded devices [10]. YOLOv8-PigLite achieves a significant reduction in parameters and computation through structural optimizations such as dual-branch bottlenecks, grouped convolutions and BiFPN, and maintains high mAP and FPS in behavior detection tasks on multiple datasets, highlighting the application potential of ultra-lightweight models in pig behavior recognition scenarios [11]. However, three key limitations remain. (1) The first is over-reliance on the previous generation of frameworks (YOLOv5/8), though YOLO11n, the latest state-of-the-art lightweight detector, was selected in this study instead of the widely used YOLOv8n. According to the official benchmark from Ultralytics [12] and our preliminary experiments, YOLO11n consistently outperforms YOLOv8n across key metrics: it achieves a higher mAP0.5 (0.912 vs. 0.805) and mAP@0.5:0.95 (0.696 vs. 0.617), with fewer parameters (2.59 M vs. 3.01 M) and lower GFLOPs (6.4 vs. 8.1). This superior accuracy–efficiency balance makes YOLO11n more suitable for real-time, lightweight pig behavior detection in complex farming environments [13]. (2) Single attention/feature fusion modules struggle to balance multi-scale modeling, cross-scale information flow, and computational efficiency [14]. (3) Simplified transfer learning and ablation experiment designs [15]. To specifically tackle these challenges, we selected three modules based on their complementary strengths for livestock monitoring. First, SCSA-CBAM was adopted to suppress background noise and enhance fine-grained features (e.g., limbs and heads), which is critical for distinguishing pigs from similar-colored facilities. Second, WFU was integrated to dynamically fuse multi-scale features, addressing the significant scale variations of pigs at varying distances from the camera. Finally, WTConv was employed to capture long-range dependencies with linear complexity, ensuring real-time performance while maintaining global context for occluded targets. These modules form the core of our proposed framework, balancing accuracy and efficiency for edge deployment.

To fill these research gaps, the present work combines recognition precision, inference efficiency, and deployment feasibility for multi-category pig behavior identification in complex farming environments. The main contributions of this study are summarized as follows. (1) It proposes an enhanced YOLO11n-based architecture integrated with SCSA-CBAM, WFU, and WTConv modules to strengthen feature discrimination, dynamic feature fusion, and improve computational efficiency. (2) It designs a two-stage transfer learning strategy that proceeds from fine-tuning with a frozen backbone to full-network joint fine-tuning, so as to balance the convergence speed and generalization ability with limited labeled data. (3) It performs systematic ablation experiments and comparisons with mainstream detectors (e.g., YOLOv8n and Faster R-CNN) to verify the effectiveness of the accuracy–efficiency trade-off. (4) It conducts supplementary benchmarking experiments on parameters, GFLOPs, and FPS across different computing platforms, providing practical deployment guidelines for real farming applications.

## 2. Materials and Methods

### 2.1. Dataset Construction

To ensure diversity in farming conditions, pig sizes, and behavioral contexts, we developed a self-built six-category pig behavior dataset based on public datasets by combining three existing sources. The experimental images were derived from these three datasets with different formats: Dataset A is a static image library, from which valid samples were directly filtered; Datasets B and C are video datasets, from which images were randomly extracted from original video frames. All images were filtered to remove invalid samples (e.g., blurry, occluded, or target-free images) and cover diverse breeding scenarios and lighting conditions.To prevent data leakage between sets, we adopted different splitting strategies based on the data modality. For Dataset A (images), samples were randomly split at the image level. For Datasets B and C (videos), we performed the split at the video level. The key information for each dataset is summarized in Table 1, encompassing six target pig behaviors with distinct spatiotemporal features to enhance the model’s generalization and robustness in practical farming scenarios. The annotation process was conducted by two authors of this paper. We established a strict labeling protocol based on six predefined behaviors to ensure consistency. To guarantee data quality, we implemented a cross-checking mechanism: after the initial annotation, the data were reviewed by the other author. Any discrepancies or ambiguous cases were resolved through discussion until a consensus was reached. This rigorous validation process ensures the reliability of the ground truth labels.

Specifically, Dataset A captures medium-to-large and small pigs from 2–3 m heights at pens sides, covering feeding and resting behaviors. Dataset B was collected in a UK research pen (eight growing pigs) via an Intel RealSense D435i camera (1280 × 720, 6 fps) during daytime (7 AM–7 PM) over 6 weeks. Dataset C supplements group-housed juvenile piglet scenarios, enriching small-target behavioral diversity [16].

**Figure 1:**
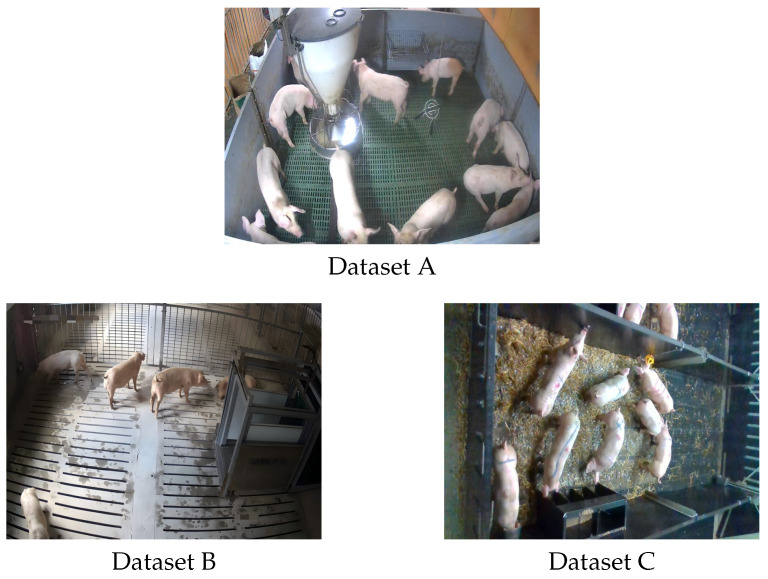
Examples of datasets.

Data collection spanned 7 AM–7 PM, including peak activity periods (09:00–11:00 and 15:00–17:00) and quiet phases, ensuring the comprehensive coverage of active (standing, walking, eating, and drinking) and resting (lateral and prone lying) behaviors to avoid sample bias [17]. Examples of the datasets are shown in Figure 1. In total, 2480 original images were obtained from the three public datasets as the source data. The unit of analysis is the individual pig, i.e., each annotated bounding box represents one pig instance, rather than one image. All the behavioral statistics are based on the number of annotated individual instances, not the number of images.

Six core behaviors (stand, walk, side, lie, eat, and drink [18]) were defined as the detection targets (detailed criteria in Table 2). A total of 2480 high-quality, behaviorally representative images were selected from the three datasets, manually annotated with LabelImg [19] using bounding boxes. The annotation information was stored in YOLO-compatible text files format for training framework compatibility. The six behaviors categories are mutually exclusive in annotation: each individual pig is assigned only one dominant behavior per image. Contextual behaviors (eating and drinking) take precedence over postural behaviors (standing and lying), ensuring no annotation overlap or ambiguity.

### 2.2. Data Augmentation

**Figure 2:**
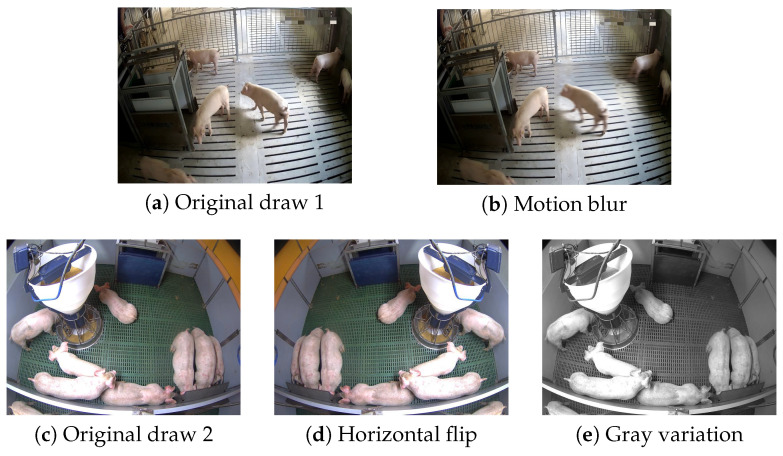
Examples of data augmentation effects.

In order to improve the model’s generalization ability and robustness in complex livestock farming environments, mitigate overfitting [20], and address the class imbalance issue related to the minority ’walking’ category, three targeted data augmentation methods were employed and data augmentation was applied only to the training set after the train/validation split (80%/20%). These methods comprised horizontal flipping, brightness adjustment, and motion blur—specifically for augmenting the ‘walk’ category—with representative augmented results illustrated in Figure 2.

**Figure 3:**
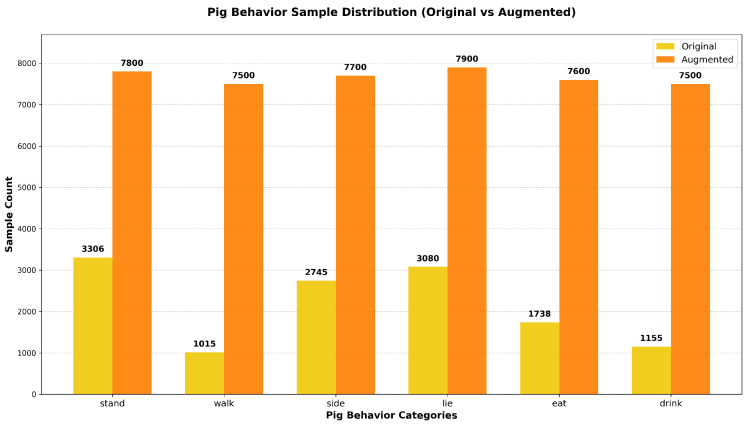
Distribution of the number of annotated individual pig instances per behavior category (original vs. augmented).

The target number of augmented instances was determined adaptively based on the original class distribution: classes with fewer initial instances (walk, drink, and eat) received stronger augmentation, while those with more instances (stand, lie, and side) were moderately supplemented. This augmentation process produced a large number of extra samples, increasing the final dataset to 7800 stand instances, 7500 walk instances, 7700 side instances, 7900 lie instances, 7600 eat instances, and 7500 drink instances. Figure 3 shows the distribution of these annotated individual instances across six behavioral categories, not the number of images. A comparison of the sample numbers between the original and augmented datasets for all six pig behavior classes is displayed in Figure 3, which clearly demonstrates that class imbalance has been effectively mitigated after the targeted data augmentation.

To guarantee consistent class distribution across each subset and the reliable evaluation of the model’s performance, the augmented dataset was divided into training and validation sets with an 8:2 ratio via stratified random sampling, yielding 13,189 training images and 2668 validation images. Comparative analysis shows that data augmentation improved mAP0.5 by 7.6% (from 0.836 to 0.912), confirming its effectiveness in mitigating class imbalance.

### 2.3. Baseline Model and Improved Architecture

Based on the YOLO11n framework, our method addresses three key issues—namely inadequate multi-scale feature modeling, conventional attention and feature fusion, and difficulty in deployment on resource-constrained edge devices—by building a multi-attention-enhanced detection network composed of SCSA-CBAM [21], WFU [22], and WTConv [23]. This network enables efficient recognition and the real-time inference of six behavioral types in complex pig farming environments. While our design retains the advantages of YOLO11n’s native efficient backbone and C3k2 structure, it further reduces computational complexity and enhances feature discriminability through cross-scale explicit attention modeling and frequency domain convolution reconstruction.

#### 2.3.1. Overall Framework and Design Motivation of C3k2 Backbone

This study adopts YOLO11n as the baseline, leveraging its backbone with C3k2 and SPPF structures for real-time performance and strong multi-scale feature extraction. However, it faces limitations in complex pig farming environments—including intense light fluctuations, occlusions, and multi-individual mixing—such as insufficient feature representation for complex backgrounds, fine-grained behavior differentiation, and small object recognition. Traditional single convolutional stacking and standard FPN-PAN fusion [24] fail to balance local textures, global context, and cross-layer semantic consistency, hindering the distinction of visually similar behaviors (e.g., standing vs. eating and prone vs. lateral lying).

**Figure 4:**
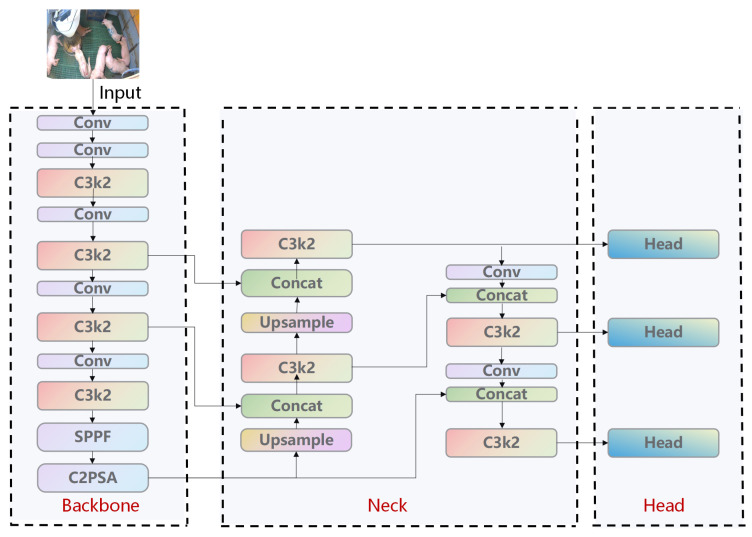
Original YOLO11n framework.

As illustrated in Figure 4, YOLO11n’s backbone consists of an initial convolutional layer, stacked C3k2 layers, and SPPF to generate multi-scale feature maps [25]. Its neck uses FPN-PAN for the bidirectional fusion of high-level semantics and low-level details [26], while the head adopts anchor-free decoding to maintain compatibility with YOLO11n’s annotation format and inference logic [27]. Although the C3k2 module outperforms its predecessor C2f in parameter efficiency and speed, its attention modeling relies on implicit convolutional selection, limiting the explicit modeling of complex backgrounds and fine-grained pose variations and often leading to misclassification of similar behaviors [28]. Thus, the introduction of refined spatiotemporal attention and frequency domain convolution is necessary.

**Figure 5:**
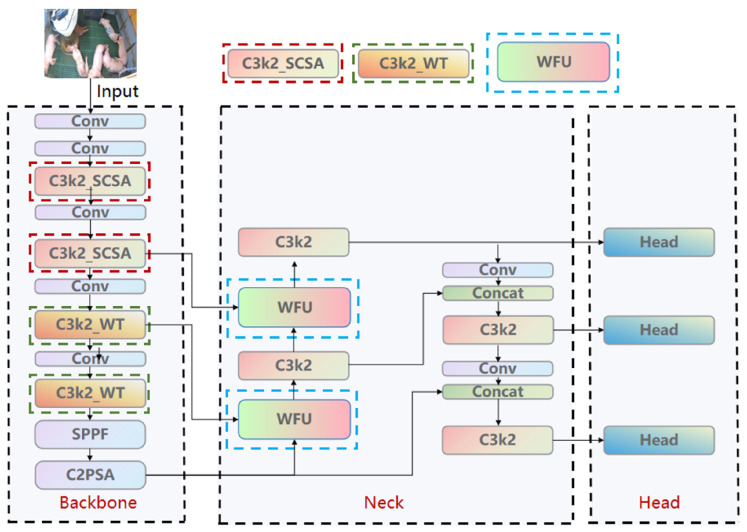
Proposed YOLO11n-SCSA-WFU-WT framework.

To tackle these problems, three lightweight improvement modules are incorporated into YOLO11n while maintaining its original core structure (Figure 5): (1) SCSA-CBAM [21] is inserted into the C3k2 bottleneck layers to enhance explicit spatial-channel recalibration; (2) WFU [22] is applied in the neck to achieve adaptive weighted fusion of multi-scale features; and (3) WTConv [23] replaces some convolutions in the backbone to expand the receptive field through frequency domain approximation (see Table 3). Following the philosophy of “minimal modification, maximum capability”, the enhanced network preserves the structural merits and pre-trained weights of YOLO11n, thus ensuring high compatibility for transfer learning and practical deployment.

#### 2.3.2. Spatial Cross-Scale Attention-CBAM Module (SCSA-CBAM)

**Figure 6:**
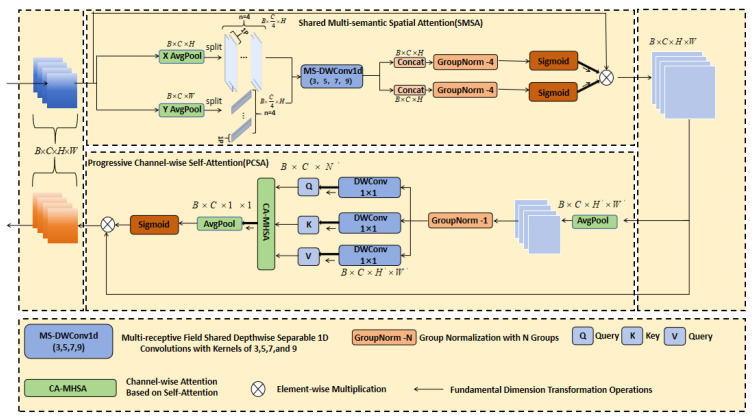
Structure of the Spatial Cross-Scale Attention-CBAM (SCSA-CBAM) module.

To enhance the backbone’s modeling capability for multi-scale behavioral patterns and complex backgrounds, the Spatial Cross-Scale Attention–CBAM (SCSA-CBAM) module is embedded in selected C3k2 bottleneck layers, performing explicit channel and spatial recalibration on intermediate features (Figure 6). The module comprises sequentially connected Channel Attention (CA) and Spatial Attention (SA), where CA determines “which features to select” while SA specifies “where to focus attention,” with low computational overhead suitable for insertion into various convolutional blocks.

Given input features $X\in R^{C\times H\times W}$, Channel Attention first obtains one-dimensional channel descriptors through global average pooling and max pooling, as shown in Equations (1) and (2):

(1) $$z_{\mathrm{avg}}\left(c\right)=\frac{1}{HW}\sum_{i=1}^{H}\sum_{j=1}^{W}X\left(c,i,j\right)$$

(2) $$z_{\max}\left(c\right)=\max_{\begin{array}{c} 1\leq i\leq H\\ 1\leq j\leq W \end{array}}X\left(c,i,j\right)$$

Then, the channel weights are obtained via shared MLP, as shown in Equation (3):

(3) $$M_{c}=\mathrm{sigmoid}\left(\mathrm{MLP}\left(z_{\mathrm{avg}}\right)+\mathrm{MLP}\left(z_{\max}\right)\right),\;M_{c}\in R^{C\times 1\times 1}$$

Obtain the channel-enhanced feature $X^{\prime }$, with the specific operation as per Equation (4):

(4) $$X^{\prime }=M_{c}⊙X$$

Here, σ(·) denotes the sigmoid function, and ⊙ represents element-wise multiplication.

In the Spatial Attention branch, $X^{\prime }$ is aggregated along the channel dimension to yield $\tilde{X}\in R^{1\times H\times W}$. This is then stacked along the channel dimension via average pooling and max pooling, combined with a Sobel operator to extract edge responses $\tilde{G}$, as shown in Equation (5):

(5) $$\tilde{G}={\left(S_{x}*\tilde{X}\right)}^{2}+{\left(S_{y}*\tilde{X}\right)}^{2}$$

Subsequently, the multi-scale pooling results of $\tilde{X}$ are concatenated with $\tilde{G}$. Following the convolution and application of the Sigmoid function, a two-dimensional Spatial Attention map $M_{s}\in R^{1\times H\times W}$ is obtained, as shown in Equation (6). The final output is denoted as *Y*:

(6) $$Y=M_{s}⊙X_{p}$$

Explicitly enhance critical information areas.

Building on CBAM, cross-scale augmentation is performed for pig behavior recognition: shared and interactive attention maps are introduced between the C3k2 outputs of adjacent stages, enabling high-resolution layers to leverage global semantics from low-resolution layers and vice versa, forming Spatial Cross-Scale Attention (SCSA). Inserted near the backbone’s P2, P3, P4, and P5 outputs, SCSA-CBAM conducts fine-grained feature selection at each stage, suppressing responses to complex backgrounds (e.g., farming facilities and feeding equipment) while enhancing behavior-related features (e.g., pig body contours, head and neck regions, and limb postures) and thus improving the separability of similar behaviors (e.g., standing vs. eating and prone vs. lateral lying) in the feature space [29].

#### 2.3.3. Weighted Feature Fusion Unit (WFU)

To address the limitations of traditional FPN-PAN—relying on simple element-wise addition and concatenation that fails to adaptively distinguish multi-scale features’ contribution to prediction [30]—the Weighted Feature Fusion Unit (WFU) is integrated into the neck. At each multi-branch fusion node, the WFU assigns learnable weights to features from different scales and paths, performing weighted summation subject to normalization constraints to explicitly model the importance of each scale’s features in the input context.

Suppose a fusion node receives features ${\left\{F_{k}\right\}}_{k=1}^{K}$ from *K* scales or paths, with corresponding learnable scalars ${\left\{a_{k}\right\}}_{k=1}^{K}$. As per Equation (7), the WFU first obtains the fusion weights via Softmax normalization:

(7) $$w_{k}=\frac{\exp(a_{k})}{\sum_{j=1}^{K}\exp\left(a_{j}\right)},\;k=1,\ldots ,K$$

The final fusion feature is expressed as formula (8):

(8) $$\hat{F}=\sum_{k=1}^{K}\left(w_{k}*F_{k}\right)$$

**Figure 7:**
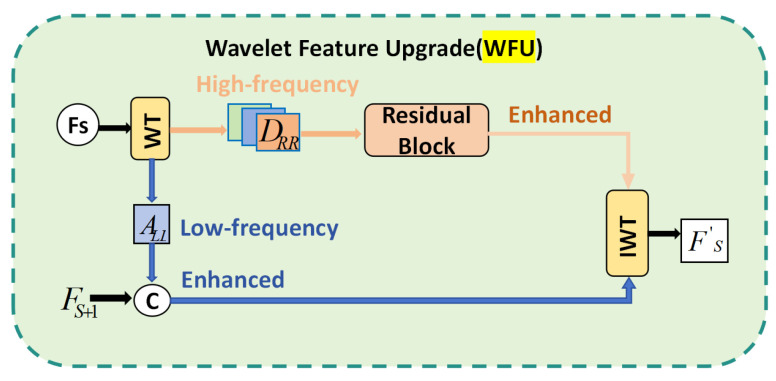
Structure of the Weighted Feature Fusion Unit (WFU).

As illustrated in Figure 7, for pig behavior recognition, small-scale features are sensitive to localized actions (e.g., eating and limb bending), while large-scale features represent overall posture and spatial relationships between individuals. The WFU enables the network to autonomously learn optimal fusion ratios across behavioral categories, camera distances, and lighting conditions during training, avoiding critical information dilution from “average fusion” while maintaining a low parameter count and computational cost (GFLOPs).

#### 2.3.4. Wavelet Transform Convolution Module (WTConv)

**Figure 8:**
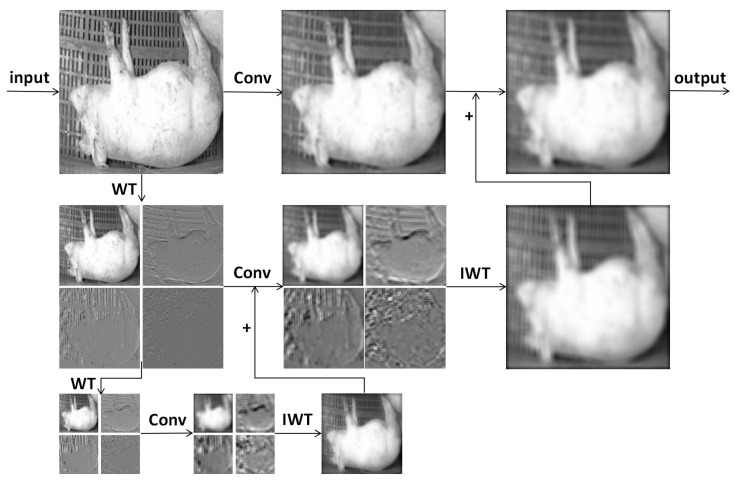
Structure of the Wavelet Transform Convolution (WTConv) module.

Traditional convolutions perform local weighting in fixed spatial neighborhoods, relying on depth stacking or dilated convolutions to expand the receptive field. However, in computationally constrained lightweight detectors, this expansion is limited, leading to the insufficient modeling of long-range dependencies and fine-grained textures [31]. To address this, we introduce the Wavelet Transform Convolution (WTConv) module and its enhanced variant WTConv into specific convolutional layers in the backbone, combining multi-resolution analysis with sub-band transformation to achieve multi-frequency feature decomposition and reconstruction (Figure 8).

Let the input feature be $F\in R^{C\times H\times W}$. WTConv employs a Discrete Wavelet Transform (DWT) to decompose the feature map into one low-frequency component (*L*) and three high-frequency sub-bands ($H^{\left(s\right)}$, where s∈{LH,HL,HH}), as shown in Equation (9).

(9) $$L,H^{\left(s\right)}=\mathrm{DWT}\left(F\right)$$

As demonstrated in Equations (10) and (11), the low-frequency and high-frequency sub-bands are processed independently [32]:

(10) $$L_{t},H_{t}^{\left(s\right)}=\mathrm{DWT}\left(W_{t}\right)$$

(11) $${\hat{L}}_{t},{\hat{H}}_{t}^{\left(s\right)}=g_{\theta }\left(L_{t},H_{t}^{\left(s\right)}\right)$$

Finally, the processed sub-bands are reconstructed via an Inverse Wavelet Transform (IWT), as shown in Equation (12):

(12) $$Y=\mathrm{IWT}(\hat{L},{\hat{H}}^{\left(s\right)})$$

This module significantly expands and enriches frequency domain information without substantially increasing the number of channels. Compared to directly employing large convolution kernels, WTConv reduces the computational complexity from approximately $O(HWk^{2})$ to $O(TM^{2}k^{2})$, effectively lowering overall GFLOPs when M≪min(H,W).

WTConv is mainly applied to low-resolution, high-semantic deep backbone layers (e.g., P4 and P5), replacing some C3k2 modules with C3k2-WTConv to enhance contour and postural geometry representation. Shallow layers (P2 and P3) retain standard convolutions to avoid the excessive smoothing of fine-grained textures. For behaviors dependent on contour and relative height (e.g., prone and lateral lying, walking), its multi-frequency representation preserves critical edges and structural details while suppressing high-frequency noise-induced false detections—all within YOLO11n’s lightweight constraints of acceptable parameters and inference latency.

### 2.4. Training and Transfer Learning Strategy

To balance convergence speed and generalization under limited labeled pig behavior data, this study adopts a “COCO pre-training + two-stage transfer learning” workflow [33]. First, the backbone, neck, and head layers are initialized with COCO pre-trained weights of YOLO11n, leveraging fundamental visual features and multi-scale structural information from general object detection [34]. During fine-tuning on the behavioral dataset, the first ten layers are frozen with a learning rate of $2\times 10^{-4}$ for five epochs, enabling rapid adaptation to pigs’ behavioral categories and annotation distributions without disrupting underlying features, thus mitigating catastrophic forgetting [35].

In Stage 2, all layers are unfrozen, maintaining the same initial learning rate of $2\times 10^{-4}$. Subsequently, the entire network undergoes 150 epochs of joint fine-tuning, incorporating cosine annealing learning rate scheduling, hierarchical learning rate decay, weight decay, and moderate Dropout. These strategies enable the SCSA-CBAM, WFU, and WTConv modules to fully learn discriminative features in pig farming environments while avoiding overfitting. Data augmentation techniques (motion blur, flipping, and brightness adjustment) are applied to simulate real-world factors (camera shake, angle variations, and lighting fluctuations), which enhances the model’s generalization and lays a solid foundation for stable deployment on server GPUss, edge boxes, and embedded terminals.

### 2.5. Experimental Parameter Settings

The proposed YOLO11n-SCSA-WFU-WT model is evaluated on a laptop equipped with a 13th-Gen Intel Core i7-13700H CPU (2.40 GHz), NVIDIA GeForce RTX 4060 Laptop GPU (8 GB VRAM), and 16 GB DDR4 RAM (4800 MT/s). The software environment is configured using Conda, including a 64-bit Windows 11 operating system, CUDA 11.6, Python 3.10.18, and the PyTorch 2.5.1 deep learning framework. This integrated hardware and software configuration ensures stable, consistent performance and compatibility with the model’s dependencies, enabling the efficient execution of training, validation, and inference tasks. The detailed training hyperparameters are provided in Table 4.

### 2.6. Evaluation Metrics

To fully and rigorously evaluate the performance of the proposed improved model, in this work, two categories of evaluation indicators are used for the YOLO11n model: accuracy metrics and efficiency metrics. These indicators will be used to measure the model’s ability with regard to object detection accuracy, generalization performance, computational cost, resource consumption, etc., thereby achieving a comprehensive evaluation of its practicality in complex pig farming environments.

#### 2.6.1. Accuracy Metrics

Accuracy metrics are established to assess the model’s ability to accurately recognize and localize target pig behaviors, with the core indicators defined as follows:

- **Precision (P)**: The proportion of true positive results out of all positive cases identified by the model; it measures the model’s ability to control false alarms. The calculation formula is
  (13) $$P=\frac{TP}{TP+FP}.$$
  TP (true positive) denotes behavioral instances where the predicted bounding box has IoU >0.5 with the ground truth box and the behavior label is identical. FP (false positive) denotes wrong detections, including incorrect labels, IoU ≤0.5, or background regions misclassified as target pigs.
- **Recall (R)**: The proportion of true positive results among all actual target samples, which evaluates whether the model can cover target behaviors without missing any. The calculation formula is
  (14) $$R=\frac{TP}{TP+FN}$$
  FN represents the number of target behavior instances that the model fails to identify.
- **Average Precision (AP)**: A combination of precision and recall at all confidence thresholds; the area under the precision–recall (PR) curve. It provides an overall evaluation index for the model’s detection performance of a single behavior category, with the following calculation formula:
  (15) $$AP=\int_{0}^{1}P\left(R\right)dR$$
  where P(R) represents the precision corresponding to the given recall value *R*.
- **Mean Average Precision (mAP@0.5)**: This metric sets the Intersection over Union at a 0.5 fixed threshold (spatial overlap ratio between the predicted and ground truth behavior bounding boxes). First, calculate the $AP_{i}$ (based on IoU = 0.5) for each of the *N* pig behavior categories; then, compute the arithmetic mean of these $AP_{i}$ values, using the following formula:
  (16) $$\mathrm{mAP}@0.5=\frac{1}{N}\sum_{i=1}^{N}{\mathrm{AP}}_{i,\mathrm{IoU}=0.5}$$
  where *N* is the total number of behavior categories (six in this study), and ${\mathrm{AP}}_{i,\mathrm{IoU}=0.5}$ is the AP of the *i*-th category when IoU = 0.5. This is the model’s mean average error in terms of the overall detection accuracy under the typical requirement for standard localisation.
- **mAP@0.5:0.95**: A measure of the model’s robustness under different levels of localisation error. First, calculate the mAP (according to the formula above) at ten consecutive IoU thresholds: 0.5,0.55,0.6,…,0.95 (incrementing by 0.05). Then, take the arithmetic mean of these ten mAP values; the calculation formula is as follows:
  (17) $$\mathrm{mAP}@0.5:0.95=\frac{1}{T}\sum_{t=1}^{T}{\mathrm{mAP}}_{t}$$
  where T=10, and ${\mathrm{mAP}}_{t}$ represents the mAP corresponding to the *t*-th IoU threshold. This indicator can be used to express the consistency of performance across various space overlap conditions in full quantity.

#### 2.6.2. Efficiency Metrics

In terms of efficiency indicators, attention should be paid to the model’s computation and storage overhead to meet the requirements for deployment on resource-constrained edge devices in farms.

- **Parameters (M)**: Measures the total number of trainable parameters in the model, directly reflecting the model’s storage requirements and memory footprint. Fewer parameters indicate better suitability for lightweight deployment.
- **GFLOPs**: Represents giga floating point operations per forward pass, quantifying the model’s computing intensity. A lower GFLOPs value indicates that the model requires fewer computations, thus reducing the hardware costs during operation.
- **Frames Per Second (FPS)**: This is the number of frames processed by the model in one second, and it is an index used to evaluate the real-time performance of the model’s inference. The larger the FPS value is, the more it satisfies the demand of real-time behavior observation in the farm.
- **Model Size (MB)**: The amount of storage space occupied by the trained model file. A smaller model size helps to transmit and deploys more quickly on edge computing devices with limited storage capacity. Both of these include but are not limited to the precision and efficiency parameters to obtain an overall grade that represents the comprehensive capacity of such a system.

The proposed model is feasible for practical applications as intelligent pig farm management needs to have balanced detection performance and implementation feasibility.

## 3. Results

### 3.1. Ablation Experiment Results

To quantitatively analyze the performance contribution of each enhancement module (SCSA, WFU, and WTConv) and their synergistic effects, as shown in Table 5, the detailed ablation experiments are presented with module configurations (marked by 🗸 for “included” and × for “not included”) and key performance metrics.

The ablation results are analyzed from three key perspectives: independent module contribution, multi-module synergy, and the balance between accuracy and lightweightness. For individual module performance: SCSA raises Precision (P) to 0.860 and improves mAP@0.5 by 1.6% (from 0.912 to 0.928), while slightly reducing parameters (from 2.59 M to 2.55 M) and GFLOPs. This demonstrates its ability to enhance feature discrimination for standing and walking behaviors, and to optimize model efficiency. WTConv achieves the most significant single-module accuracy gain, with P increasing from 0.852 to 0.909 and mAP@0.5 from 0.912 to 0.956, confirming its effectiveness in expanding receptive fields to capture fine-grained contours such as lying and lateral lying. WFU increases P to 0.912 and mAP@0.5 to 0.959, but also raises parameters (from 2.59 M to 3.59 M) and GFLOPs (from 6.4 to 8.1) due to its learnable cross-scale fusion weights. For multi-module synergy, SCSA + WFU boosts P to 0.925 and mAP@0.5 to 0.962 (vs. the single WFU) with slightly reduced parameters (3.59 M to 3.57 M), as SCSA suppresses redundant features; WFU+WT achieves P = 0.931 and mAP@0.5 = 0.971 with fewer parameters (3.42 M) than single WFU, thanks to WT’s receptive field compensation; and the full SCSA+WFU+WT combination reaches optimal performance (P = 0.937, mAP@0.5 = 0.974, and mAP@0.5-0.95 = 0.785) via complementary functions (SCSA: cross-scale attention, WFU: dynamic fusion, and WT: receptive field expansion). Regarding the accuracy–lightweight balance, compared to single WFU, the full-module model reduces parameters by 0.19 M and GFLOPs by 0.3 (via SCSA and WTConv optimization), with a 7.1 MB size (only 1.7 MB larger than baseline), which satisfies the <10 MB deployment requirement for edge devices.

### 3.2. Recognition Performance for Each Behavior Category

To comprehensively quantify the performance improvement of the enhanced model over the baseline, this section focuses on key metrics including the baseline mAP@0.5, enhanced mAP@0.5, mAP@0.5 improvement, mAP@0.0-0.95 improvement rate, Precision (P) improvement rate, Recall (R) improvement rate, and enhanced F1-score. To ensure fair comparison, all the models are trained under identical conditions, including the same dataset split, data augmentation, training epochs, and input resolution. The detailed comparison results are summarized in Table 6, and a detailed in-depth analysis of these results is provided below.

The enhanced YOLO11n-SCSA-WFU-WT model achieves significant and balanced improvements: the overall mAP@0.5 increases by 6.2% (0.912 to 0.97), mAP@0.5-0.95 by 12.8%, Precision by 8.5%, Recall by 10.3%, and the F1-score reaches 0.936. This demonstrates that the integrated SCSA-CBAM, WFU, and WTConv modules effectively address the baseline’s limitations in feature discrimination, cross-scale fusion, and receptive field coverage. Notably, side (lateral lying) and drink (drinking) show the most prominent gains: side’s mAP@0.5 rises by 8.4% (the highest) and Precision by 16.3% (aided by SCSA-CBAM and WTConv reducing misclassification), while drink’s mAP@0.5-0.95 increases by 18.0% (the highest) and Recall by 14.1% (WFU strengthens small-target association). Lie and eat maintain high performance (mAP@0.5 ≥ 0.974, F1 ≥ 0.936), stand achieves balanced improvements (F1 = 0.932), and walk—though with the lowest mAP@0.5-0.95 gain (3.2%) due to dynamic features and limited samples—still meets the practical requirements (mAP@0.5 = 0.970, F1 = 0.920).

The model presents two key advantages for intelligent pig farming: First, it attains high and balanced performance over all six core behaviors (mAP@0.5 ≥ 0.970 and F1 improvement ≥ 4.6%), ensuring that no health- or welfare-related behaviors are overlooked. Second, it performs well in small-target (drinking) and fine-grained recognition (side/lie) scenarios, which are major pain points in complex pig farming environments, thus verifying the practical value of the integrated module design.

**Figure 9:**
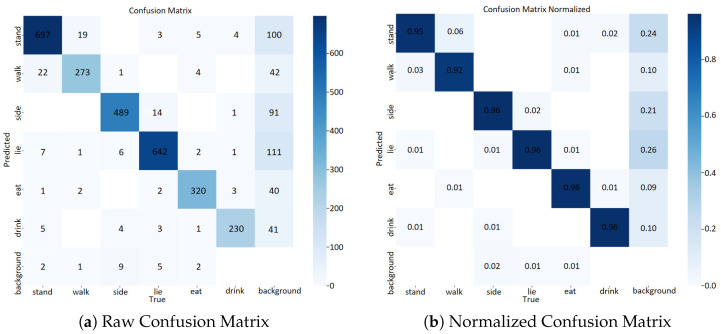
Confusion matrix results of the proposed model (raw count and normalized proportion) for six pig behavior categories and backgrounds.

Confusion matrices are used to evaluate the model’s classification performance [36] across six pig behavior categories and backgrounds, and the results are plotted in Figure 9 (detection parameters are detailed in Table 4). The left raw confusion matrix shows that the diagonal elements (correct classifications) are significantly larger than the off-diagonal ones; for example, 697 “stand” and 642 “lie” samples are correctly classified, with minor misclassifications. Thus, it can be determined that the model has distinguished most behaviors accurately. The right normalized confusion matrix shows that all diagonal elements are ≥0.95 and off-diagonal elements are <0.06, indicating that there is almost no misclassification of behaviors and background, which confirms the high category-specific accuracy and minimal inter-class confusion of the model. Performance: It can recognize the six pig behaviors accurately, effectively distinguish them from the background, and maintain a low inter-class misclassification rate; therefore, it is robust enough for practical use.

### 3.3. Comparative Experiments with Mainstream Detectors

In order to further confirm the superiority of the proposed YOLO11n-SCSA-WFU-WT model in terms of its accuracy, efficiency, and deployment feasibility, we conduct comparative experiments with four mainstream detectors (YOLO11s, YOLOv8n, YOLOv26n [37,38], and Faster R-CNN) on the same pig behavior dataset. The comparison focuses on core metrics including detection accuracy, model lightweight, and real-time performance. The detailed comparison results are summarized in Table 7, and a comprehensive analysis follows.

YOLO11s achieves the highest accuracy (mAP@0.5 = 0.978; mAP@0.5-0.95 = 0.832) but introduces high computational overhead (9.43 M parameters; 21.6 GFLOPs), which restricts its deployment on edge devices. Our proposed model obtains an mAP@0.5 of 0.974 (only 0.004 lower than YOLO11s) and mAP@0.5-0.95 = 0.785, and performs significantly better than Faster R-CNN, YOLOv8n, and YOLOv26n, which verifies the effectiveness of the integrated SCSA-CBAM, WFU, and WTConv modules.

Faster-RCNN achieves moderate accuracy (mAP50 = 0.915) but involves extremely high computational complexity (43.28 M parameters; 280.82 GFLOPs) and a slow inference speed (4.68 FPS), making it unsuitable for real-time monitoring. YOLOv8n maintains a good balance between lightweight property (3.01 M parameters; 6.2 MB) and detection accuracy, but it lacks explicit attention mechanisms, resulting in performance degradation in complex farm environments [39]. YOLOv26n is ultra-lightweight (2.51 M parameters; 5.4 MB) and runs at a high speed of 81.21 FPS, yet it sacrifices detection accuracy (mAP50 = 0.733) due to its limited feature extraction ability.

In terms of lightweight performance, the proposed model achieves a favorable trade-off: its parameters (3.40 M), GFLOPs (7.8), and model size (7.1 MB) are much lower than those of YOLO11s and Faster-RCNN, while only being slightly higher than YOLOv8n and YOLOv26n. Its inference speed reaches 72.28 FPS, which fully meets the real-time demand (≥30 FPS) and supports stable monitoring for group-housed pigs. Notably, it maintains a stable performance for small-target (drinking) and fine-grained (side/lie) behaviors in complex environments, and is suitable for edge device deployment (e.g., embedded terminals).

**Figure 10:**
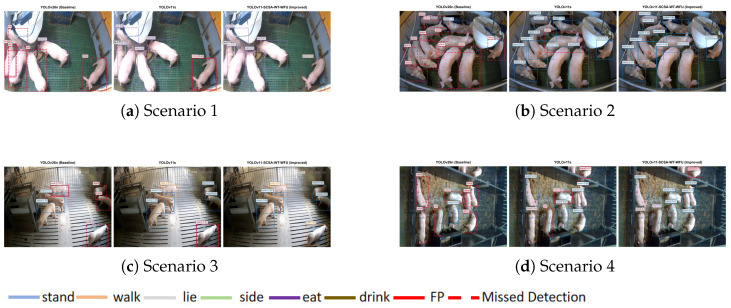
Detection results comparison among YOLO11n-SCSA-WFU-WT, YOLO11s, and YOLOv26n.

Overall, the proposed model achieves the optimal trade-off between accuracy, lightweight design, and real-time performance, making it more suitable for practical deployment on resource-constrained edge devices in complex pig farming environments. A comparison of the detection results with YOLO11s and YOLOv26n is shown in Figure 10.

### 3.4. Impact of Two-Stage Transfer Learning on Model Performance

To validate the effectiveness of the proposed two-stage transfer learning (TL) strategy in scenarios with limited data, a controlled experiment was conducted where only the TL setup was varied (all other conditions, including the model’s architecture, hyperparameters, and the dataset, remained identical). Table 8 presents the key performance metrics of the model with two-stage TL (COCO pre-training and frozen backbone fine-tuning and joint fine-tuning) and the model trained from scratch.

The controlled experiment confirms the multi-dimensional benefits of two-stage TL: compared to the TL-free model, it increases mAP@0.5 by 1.9%, mAP@0.5-0.95 by 2.0%, and drinkAP by 2.1% (the most notable gain). This is attributed to TL reusing general visual features (edges, textures, and small-target contours) pre-trained on COCO, avoiding redundant low-level feature learning on the small-scale pig behavior dataset and enhancing small-target (e.g., drinking) recognition. Beyond accuracy, TL optimizes training efficiency (epochs required for convergence reduced from 42 to 28, 33.3% faster) and generalization ability (train/val box_loss gap reduced by 18.5%), mitigating overfitting in limited data scenarios and lowering the GPU overhead, all without sacrificing the model’s lightweight properties and real-time performance.

### 3.5. Comparative Analysis of Different Attention Mechanisms

To address the core research question of “how attention mechanism design affects pig behavior recognition performance” and provide data-driven support for the superiority of the proposed SCSA (Spatial Cross-Scale Attention) mechanism, comparative experiments between SCSA and two mainstream single-dimensional attention mechanisms (channel-wise attention SE [13] and spatial-wise attention LSKA [10,11]) are conducted by isolating the attention mechanism variable (only replacing the attention mechanism) under the same WTConv and WFU framework to ensure fair comparison. The core evaluation metrics include the detection accuracy, lightweight property, and real-time performance, with the detailed comparative results summarized in Table 9.

As shown in Table 9, the SCSA-based model achieves optimal comprehensive performance: with lightweight properties comparable to LSKA (3.4 M parameters, 7.8 GFLOPs, and 7.1 MB), it attains the highest detection accuracy (mAP@0.5 = 0.974; mAP@0.5-0.95 = 0.785) and acceptable real-time performance (72.28 FPS). In contrast, the SE-based model suffers from severe parameter redundancy (5.4 M) and poor real-time performance (38 FPS), while LSKA excels in being lightweight but lacks sufficient accuracy; the baseline model without attention mechanisms has the lowest accuracy, confirming attention-driven feature enhancement is essential for fine-grained recognition. The performance gap stems from single-dimensional attention limitations: SE ignores spatial context and LSKA fails to model channel redundancy, and both struggle with the cross-scale and fine-grained characteristics of pig behaviors [40]. SCSA integrates cross-scale channel calibration and Spatial Attention, solving the “channel–spatial decoupling” problem and forming a synergistic effect with WTConv and WFU, providing high-quality feature inputs without disrupting lightweight balance. The existing research supports this conclusion: mainstream studies confirm dual-dimensional/multi-module attention mechanisms outperform single-dimensional ones in terms of suppressing background interference and distinguishing similar behaviors, further validating SCSA’s effectiveness and practical value [41].

### 3.6. Visual Analytics

To intuitively verify the training stability, performance robustness, and feature attention modules of the proposed model, this section analyzes three types of visualizations: loss function convergence curves, precision–recall (PR) curves, and behavior detection attention region comparisons. These visualizations provide multi-dimensional evidence for the model’s advantages.

#### 3.6.1. Loss Function Convergence Curves

**Figure 11:**
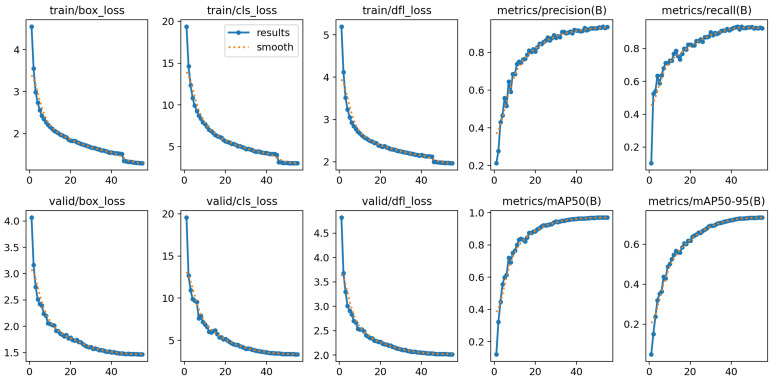
Convergence trends of training/validation losses and key metrics during model training.

Figure 11 displays the convergence trends of training/validation losses and the key metrics during model training. The three training losses drop rapidly in the first 20 epochs—for instance, train/cls_loss decreases from around 20 to about 5—and then tend to stabilize after 40 epochs. This indicates that the model quickly adapts to the pig behavior recognition task in the early training stage, and the loss saturation in the later stage indicates no overfitting [42]. Meanwhile, the validation losses show a consistent downward trend with the training losses, and the gap between training and validation losses remains small, which verifies the good generalization ability of the model. In terms of metrics, metrics like Precision, Recall, mAP@0.5, and mAP@0.5-0.95 rise rapidly in the first 20 epochs and stabilize at a high level (e.g., mAP@0.5 approaches 0.97), confirming that the model’s performance is improves and stabilizes during training.

#### 3.6.2. Precision-Recall Curves

**Figure 12:**
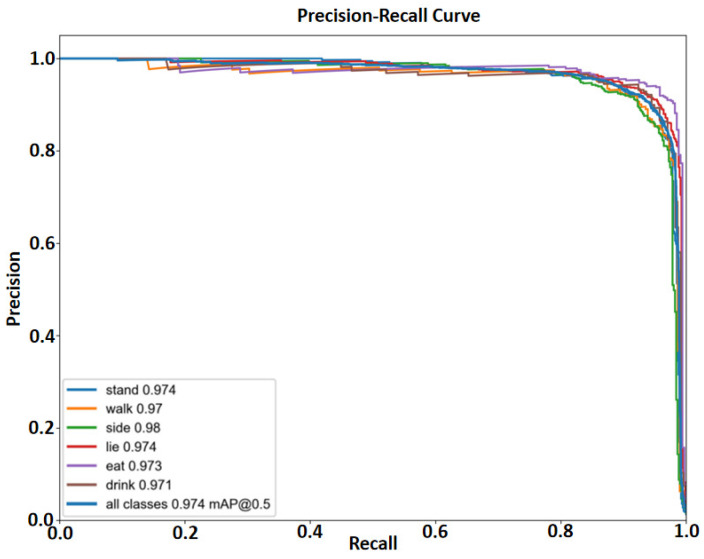
PR curves of the six pig behavior categories (stand, walk, side, lie, eat, and drink) for the proposed model.

**Figure 13:**
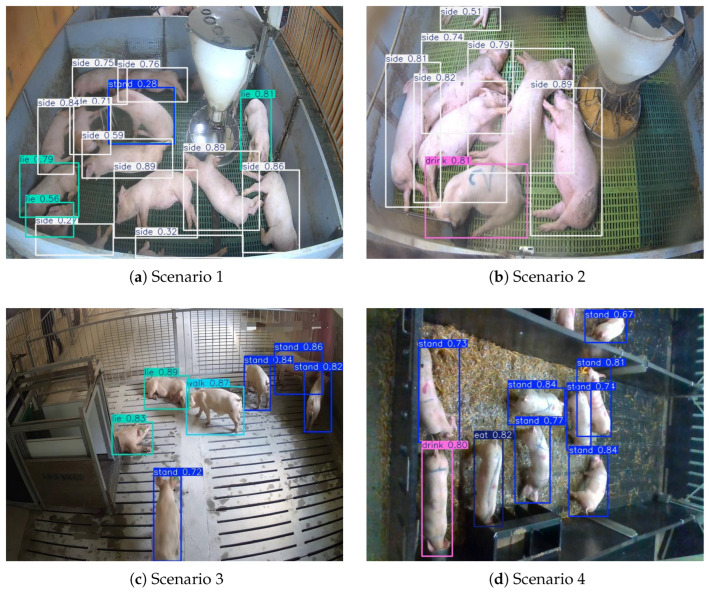
Model detection results in four different scenarios.

Figure 12 shows the PR curves of the six pig behavioral categories for the proposed model. All six behavioral categories have PR curves that are close to the upper-right corner of the graph: when Recall approaches 1.0, high Precision is maintained across most Recall ranges. For example, the side behavior has a PR curve that almost closely aligns with the top boundary, with an AP of 0.98. Moreover, the curves of different behaviors are highly concentrated, and the overall mAP@0.5 reaches 0.974. This indicates that the model maintains a good balance between Precision and Recall across all behavioral categories, reflecting strong performance robustness. Our model’s detection results are shown in Figure 13.

#### 3.6.3. Grad-CAM Heatmap Analysis

**Figure 14:**
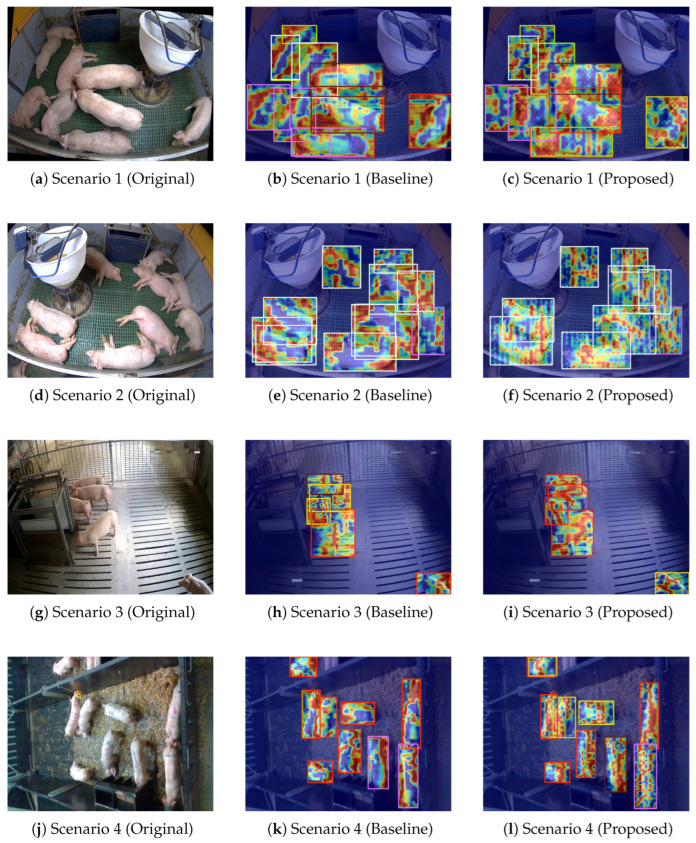
“Original image + baseline model heatmap + proposed model heatmap” comparison across four distinct pig farming scenarios.

To further visualize the model’s feature attention mechanism, Figure 14 presents the “original scene image, baseline model heatmap, and proposed model heatmap” comparison across four distinct pig farming scenarios (where red/yellow regions in heatmaps indicate high attention to key target regions, and blue/purple regions indicate low attention to background or interference) [43]. In Scenario 1, the baseline model’s heatmap spreads attention to the ground and surrounding facilities, while the proposed model’s heatmap concentrates continuously on pig bodies with minimal background activation; in Scenario 2, the baseline heatmap involves both pigs and equipment, whereas the proposed model strictly focuses attention on pig contours to avoid surrounding interference; in Scenario 3, the baseline heatmap scatters attention to the slatted floor, while the proposed model focuses on moving pigs to accurately capture core targets; and in Scenario 4, the baseline heatmap spreads attention to bedding material, while the proposed model concentrates on resting pigs and suppresses surrounding bedding. The consistent performance across the four scenarios demonstrates that the proposed model effectively strengthens the ability to focus on pig targets and reduce interference from the background. This attention mechanism plays a vital role in improving detection accuracy and robustness under complex pig farming conditions.

## 4. Discussion

### 4.1. Analysis of the Effectiveness of the Improved Module

The SCSA-CBAM module enhances behavior recognition performance by cascading Channel and Spatial Attention. Channel Attention dynamically enhances the feature channels of pig body contours, limb textures, and behavioral boundaries through global aggregation and weight learning, while suppressing background noise such as manure and feeding equipment. Quantitative results validate this noise suppression capability: behaviors prone to background interference, such as ’side’ and ’lie’, achieved precision gains of 16.3% and 11.9% respectively (Table 6). Combining spatial attention with Sobel edge operators can enhance some parts of the pig image, especially its head, limbs, and key regions; this provides an “early focus” for the intermediate layer (e.g., C3k2) of this network. The ablation study further confirms that introducing SCSA alone increased the mAP@0.5-0.95 from 0.696 to 0.719 (Table 5), validating its role in prioritizing key behavioral features.

For cross-scale feature fusion, traditional FPN-PAN adopts fixed concatenation or element-wise summation, acking adaptability to different behavioral scales. The WFU provides learnable weights to different scale branches before fusion, dynamically adjusting the contribution of high-resolution detail features and low-resolution semantic features according to behavioral categories and scene complexity. This adaptability is crucial for behaviors with substantial scale differences. In the ablation study, adding WFU to the baseline increased the Recall from 0.831 to 0.918 (Table 5). This is especially helpful in dealing with behaviors that have substantial differences in scale (such as close-range eating and far-field standing), where dynamic behaviors like ’stand’ and ’walk’ saw Recall improvements of 9.9% and 10.1% (Table 6), demonstrating reduced missed detections.

WTConv replaces standard convolutions in the deeper layers of the backbone (e.g., P4 and P5). Its core idea decomposes large-scale spatial modeling into local window operations, and approximates global dependencies through local subregion interactions. This reduces computational complexity from near-quadratic growth to near-linear growth, expanding receptive fields while speeding up inference. The ablation results show that the WTConv module alone contributed the most significant single-module boost to Precision, increasing it from 0.852 to 0.909 (Table 5). Shallow layers (e.g., P2 and P3) retain standard convolutions to maintain fine-grained textures such as edges. This enhanced feature representation is particularly beneficial for interaction-heavy behaviors; for instance, ’eat’ and ’drink’ behaviors achieved the highest mAP50-95 improvements of 16.7% and 18.0% respectively (Table 6). Statistical results indicate that the model has similar complexity to YOLO11n, with comparable FPS and latency performance, incurring only marginal overhead, which demonstrates significant engineering value for lightweight design and real-time deployment.

### 4.2. The Effectiveness of Transfer Learning Strategies

Given the high annotation cost and limited sample size of pig behavior data, the proposed two-stage transfer learning strategy shows significant practical advantages. In the first stage, the backbone layers are frozen, and only the detection and classification head is fine-tuned. This makes full use of the general edge, texture, and shape features pre-trained on COCO, avoids excessive tuning of low-level features on small datasets, and greatly reduces the risk of overfitting. In the second stage, we gradually unfreeze and jointly fine-tune the whole network, so that high-level semantic and mid-level structural features can adapt to the behavioral distribution and background texture of specific pig farming environments, achieving the in-depth adaptation from general visual representation to pig-specific behavior representation. Compared with one-step global fine-tuning, this two-stage strategy brings a more stable loss curve, faster early mAP convergence, and slightly higher final accuracy, and better balances convergence speed and generalization ability under the condition of limited data.

Moreover, the original pig behavior dataset has a limited number of annotated images, making direct training prone to scene and pose distribution imbalances. This study adopts data augmentation techniques to expand the effective training sample size. This method increases the proportion of behavioral samples in different lighting, viewing angles, and mild occlusions, thereby expanding the coverage of the feature space without changing the semantic labels. Based on the training results, data augmentation enhances the model’s accuracy and recall rate in strong light, warm light, and mild occlusion situations; therefore, it is an complements the transfer learning strategy.

### 4.3. Benchmarking Analysis with Existing Work

Compared to existing YOLOv5/YOLOv8-based pig behavior recognition methods, this work has obvious advantages in both the algorithm framework and validation process. First, it uses the most advanced YOLO11n as the baseline and utilizes its improvements in decoupled head design, feature organization and engineering implementation to create an excellent speed–accuracy base. Based on this, SCSA-CBAM, WFU, and WTConv modules are combined to achieve a synergistic effect of “architectural dividends + module enhancement”. Unlike single-dimensional attention mechanisms, such as LSKA and SE, SCSA-CBAM explicitly constructs Channel and Spatial Attention distributions to better characterize complex behaviors determined by posture, contact relationships, and local texture, which exhibit better discriminability in occluded and overlapping group-rearing conditions.

In training strategy and experimental design, this work details the two-stage transfer learning workflow—including frozen layers, learning rate decay, and epoch configurations—and quantifies its impact on convergence speed and performance by comparing with single-stage global fine-tuning, providing a reproducible training paradigm. Moreover, unlike studies that only report accuracy metrics, it systematically presents multi-dimensional benchmarking results and comprehensively evaluates the accuracy–efficiency trade-off across mAP, recall, parameters, GFLOPs, and FPS, offering quantitative references for farms to select deployment options. Compared to lightweight methods such as YOLOv8-PigLite, YOLO-DLHS-P, and PAB-Mamba-YOLO, this approach prioritizes interpretability and comparability in engineering implementation.

### 4.4. Limitations and Directions for Improvement

**Figure 15:**
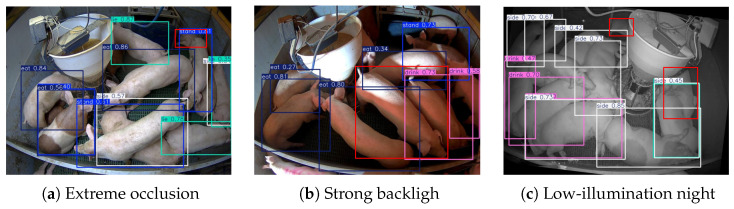
Typical missed detection cases of the proposed model.

Although the proposed model achieves a satisfactory performance on the target dataset, typical missed detections in complex pig farming environments (Figure 15) reveal critical limitations (red dashed boxes mark missed detections).

The root causes are identified as follows. (1) Under extreme occlusion (Figure 15a), SCSA fails to recalibrate fragmented features (e.g., ears and snouts), and WTConv’s local window modeling cannot capture the global structural correlation of occluded pigs. (2) In strong backlight (Figure 15b), single-modal visible light detection cannot suppress overexposure-induced pseudo-features, and WFU’s fusion weights (optimized for normal lighting) fail to enhance low-signal features. (3) At night (Figure 15c), low illumination causes high noise—WTConv’s non-specific noise suppression cannot distinguish weak pig features from background clutter, and SCSA loses the ability to identify blurred textures. Additionally, it is important to acknowledge that the current dataset is sourced from a specific pig farming environments. The absence of external validation on independent farms or diverse camera systems limits the conclusions about cross-domain generalization in broader real-world scenarios.

Future research will focus on core improvements: optimizing feature fusion in SCSA/WFU modules; integrating visible light with infrared/thermal imaging via dual-modal attention to enhance lighting robustness; and expanding datasets with extreme scenario samples and applying scenario-aware data augmentation to boost generalization. Moreover, we will improve cross-scenario generalization across pig breeds/pen types and the recognition of complex interactive behaviors (e.g., play and aggression) by validating on large-scale multi-source datasets, and will incorporate temporal information (e.g., lightweight Transformers) to extend frame-level detection to event-level recognition. Furthermore, in future research, we will explore the integration of rotatable boxes (OBB) and object segmentation methods to address the limitations of traditional detection boxes and further improve the accuracy of target localization and feature perception.

## 5. Conclusions

Based on the lightweight YOLO11n framework, this study builds a multi-module improved network to achieve multi-class pig behavior recognition, addressing core issues in complex pig farm environments such as low precision, model weight limitations and deployment restrictions, and seeks a balance between algorithmic performance and engineering practicability. The proposed model combines the SCSA-CBAM, WFU, and WTConv modules into the backbone and neck layers, supplemented by a two-stage transfer learning approach and targeted data augmentation. The experimental results on the self-built six-category pig behavior dataset show that the optimized model has achieved an mAP@0.5 higher than YOLO11n by 6.2%, and improved AP across all behavioral categories of pigs. It retains lightweight characteristics (3.40 M parameters; 7.8 GFLOPs) to meet the requirements of real-time inference and low computational power deployment. Ablation studies on the system validate the combined benefits of the multi-module design over a single-structure upgrade, and comparative experiments confirm that it has better accuracy–efficiency trade-off than mainstream lightweight detectors (such as YOLOv8n and YOLOv26n) and exhibits robust stability in situations where there are many overlapping pigs, lighting variations and scale differences. A two-stage transfer learning strategy, along with data augmentation, can facilitate the rapid convergence to a well-performing model under conditions of scarce labels; therefore, it has been proven to be practicable in the data-scarce domains. In summary, the multi-attention-enhanced model proposed in this paper provides a high-accuracy, low-latency, and low-resource-consumption unified solution that offers a deployable technical route for large-scale pig farms to achieve real-time behavior monitoring, laying the groundwork for potential downstream applications such as early disease warning and animal welfare evaluation. Future work will focus on multimodal fusion, temporal modeling, cross-scenario adaptation, and task expansion to build an end-to-end behavioral perception system for intelligent livestock farming. 

## Figures and Tables

**Table 1:** Overview of the three public pig behavior datasets.

Dataset	Breeding Scenario	Pig Size/Type	Number of Images
Dataset A	Pigsty side (2/3 m shooting heights)	Medium-large/Medium-small	880
Dataset B	SRUC (UK); Single pen (5.8 × 1.9 m)	8 growing pigs (30 kg)	848
Dataset C	Piglet group-housed pens	Juvenile piglets (4–5 per video)	752

Note: The total number of annotated individual instances (counted by pig bounding boxes, not images) across six behavioral categories: stand (3306), walk (1015), side (2745), lie (3080), eat (1738), and drink (1155).

**Table 2:** Classification criteria for six target pig behaviors.

Behavior Category	Classification Criterion
Stand	Pigs stand stably with four legs steadily on the ground, without limb crossing or lifting.
Walk	Pigs show crossed forelegs, crossed hind legs, or lifted limbs, which can be clearly distinguished in a single frame.
Side	Pigs use one side of the trunk as support, with one side of the trunk fully touching the ground and limbs extended.
Lie	Pigs have all four limbs and abdomen touching the ground simultaneously, with limbs in a bent posture and head stretched forward.
Eat	The pig’s head is fully inside the fixed feeder; this is confirmed by the presence of feed in the feeder and the pig’s proximity to the feeder.
Drink	The pig’s head is fully inside the fixed nipple drinker/water trough; this is confirmed by the presence of water and the pig’s large-angle head lowering.

**Table 3:** Insertion locations of the proposed modules in the YOLO11n architecture.

Module	Component	Insertion Location
SCSA-CBAM	Backbone	C3k2 bottleneck layers (Stage 2–4)
WTConv	Backbone	Deep C3k2 layers (P4/P5 feature maps)
WFU	Neck	FPN-PAN fusion paths

**Table 4:** Training parameters for the YOLO11n-SCSA-WFU-WT model.

Parameter	Value
Total Epochs	55
Batch Size	4
Initial Learning Rate (lr0)	$2\times 10^{-4}$
Learning Rate Schedule	Cosine Annealing
Optimizer	AdamW
Weight Decay	0.0005
Input Image Size	640 × 640
Confidence Threshold	0.25
NMS IoU Threshold	0.7

**Table 5:** Ablation experimental results of different module combinations on YOLO11n baseline (averaged over five random seeds).

Model	SCSA	WFU	WT	P	R	mAP50	mAP50-95	Params (M)	FLOPs (G)	Size (MB)
Baseline	×	×	×	0.852(0.005)	0.831(0.004)	0.912(0.003)	0.696(0.003)	2.59	6.4	5.4
+ SCSA	🗸	×	×	0.860(0.004)	0.857(0.003)	0.928(0.002)	0.719(0.002)	2.55	6.3	5.4
+ WT	×	×	🗸	0.909(0.003)	0.897(0.004)	0.956(0.002)	0.750(0.002)	2.57	6.3	5.4
+ WFU	×	🗸	×	0.912(0.002)	0.918(0.003)	0.959(0.001)	0.757(0.001)	3.59	8.1	7.6
+SCSA+WFU	🗸	🗸	×	0.925(0.002)	0.922(0.002)	0.962(0.001)	0.767(0.001)	3.57	8.1	7.5
+WFU+WT	×	🗸	🗸	0.931(0.002)	0.927(0.002)	0.971(0.001)	0.778(0.001)	3.42	8.2	7.2
+SCSA+WFU+WT	🗸	🗸	🗸	0.937(0.001)	0.934(0.002)	0.974(0.001)	0.785(0.002)	3.40	7.8	7.1

**Table 6:** Class-wise performance comparison (absolute values of enhanced model and improvements over baseline).

Behavior	Enhanced Model (Absolute)					Improvements (%)				
	P	R	F1	mAP50	mAP50-95	ΔP	ΔR	ΔF1	ΔmAP50	ΔmAP95
Stand	0.937	0.927	0.932	0.974	0.790	+7.3	+9.9	+5.7	+6.2	+13.0
Walk	0.910	0.931	0.920	0.970	0.745	+3.8	+10.1	+4.6	+5.7	+3.2
Side	0.950	0.931	0.940	0.980	0.820	+16.3	+7.2	+8.2	+8.4	+14.2
Lie	0.954	0.941	0.948	0.974	0.800	+11.9	+13.2	+9.1	+6.9	+11.8
Eat	0.954	0.928	0.941	0.975	0.789	+10.5	+7.0	+5.3	+5.6	+16.7
Drink	0.926	0.946	0.936	0.971	0.766	+10.8	+14.1	+9.8	+8.0	+18.0
All	0.937	0.934	0.936	0.974	0.785	+8.5	+10.3	+6.3	+6.2	+12.8

**Table 7:** Comparative results of mainstream detectors and the proposed model.

Model	mAP50	mAP50-95	Parameter Count (M)	GFLOPs	Inference Speed (FPS)	Model Size (MB)
Faster-RCNN	0.915	0.710	43.28	280.82	4.68	165.47
YOLO11s	0.978	0.832	9.43	21.6	48.3	19.2
YOLO11n	0.912	0.696	2.59	6.4	78.84	5.4
YOLOv8n	0.805	0.617	3.01	8.1	75.77	6.2
YOLOv26n	0.733	0.554	2.51	5.2	81.21	5.4
YOLO11n-SCSA-WFU-WT	0.974	0.785	3.40	7.8	72.28	7.1

**Table 8:** Performance comparison between models with/without two-stage transfer learning.

Metric	With Two-Stage TL	Without TL	Improvement (%)
mAP50	0.974	0.951	+1.9
mAP50-95	0.735	0.715	+2.0
drinkAP	0.971	0.950	+2.1
Stable Convergence Epoch	28	42	−33.3
Train/Val box_loss Gap	0.88	1.08	−18.5

**Table 9:** Performance comparison of different attention mechanisms under the same WT + WFU framework.

Model	mAP@0.5	mAP@0.5-0.95	Params (M)	GFLOPs	FPS	Stand AP	Model Size (MB)
YOLO11n	0.912	0.696	2.59	6.4	78.84	0.917	5.4
YOLO11n+SE+WT+WFU	0.953	0.738	5.40	9.4	38	0.936	10.8
YOLO11n+LSKA+WT+WFU	0.942	0.714	3.40	7.8	60.23	0.931	7.1
YOLO11n+SCSA+WT+WFU	0.974	0.785	3.40	7.8	72.28	0.974	7.1

## Data Availability

The datasets utilized in this study are publicly available and can be accessed via the following platforms or institutional repositories: RoboFlow Pig Behavior Dataset: Available on RoboFlow Universe at https://universe.roboflow.com/swine-tktu8/pig-behavior-8xbgn/dataset/1 (accessed on 17 December 2025); University of Edinburgh PIGDATA Dataset: Provided by the University of Edinburgh, accessible at https://homepages.inf.ed.ac.uk/rbf/PIGDATA/ (accessed on 17 December 2025); and the Kaggle Pig Video Dataset: Available on Kaggle at https://www.kaggle.com/datasets/hu233wu/pig-video (accessed on 17 December 2025). The relevant code of this study is available from the corresponding author upon reasonable request.
